# Reactively sputtered nickel nitride as electrocatalytic counter electrode for dye- and quantum dot-sensitized solar cells

**DOI:** 10.1038/srep10450

**Published:** 2015-05-21

**Authors:** Jin Soo Kang, Min-Ah Park, Jae-Yup Kim, Sun Ha Park, Dong Young Chung, Seung-Ho Yu, Jin Kim, Jongwoo Park, Jung-Woo Choi, Kyung Jae Lee, Juwon Jeong, Min Jae Ko, Kwang-Soon Ahn, Yung-Eun Sung

**Affiliations:** 1 Center for Nanoparticle Research, Institute for Basic Science (IBS), Seoul 151-742, Republic of Korea; 2 School of Chemical and Biological Engineering, Seoul National University, Seoul 151-742, Republic of Korea; 3 Department of Nanomaterials Science and Engineering, Korea University of Science and Technology, Daejeon 305-350, Republic of Korea; 4 Photo-electronic Hybrids Research Center, Korea Institute of Science and Technology (KIST), Seoul 136-791, Republic of Korea; 5 Samsung SDI Materials Devision, OLED Development Group 1, Uiwang 437-711, Republic of Korea; 6 Green School, Korea University, Seoul 136-701, Republic of Korea; 7 KU-KIST Graduate School of Converging Science and Technology, Korea University, Seoul 136-701, Republic of Korea; 8 School of Chemical Engineering, Yeungnam University, Gyeongsan 712-749, Republic of Korea

## Abstract

Nickel nitride electrodes were prepared by reactive sputtering of nickel under a N_2_ atmosphere at room temperature for application in mesoscopic dye- or quantum dot- sensitized solar cells. This facile and reliable method led to the formation of a Ni_2_N film with a cauliflower-like nanostructure and tetrahedral crystal lattice. The prepared nickel nitride electrodes exhibited an excellent chemical stability toward both iodide and polysulfide redox electrolytes. Compared to conventional Pt electrodes, the nickel nitride electrodes showed an inferior electrocatalytic activity for the iodide redox electrolyte; however, it displayed a considerably superior electrocatalytic activity for the polysulfide redox electrolyte. As a result, compared to dye-sensitized solar cells (DSCs), with a conversion efficiency (*η*) = 7.62%, and CdSe-based quantum dot-sensitized solar cells (QDSCs, *η* = 2.01%) employing Pt counter electrodes (CEs), the nickel nitride CEs exhibited a lower conversion efficiency (*η* = 3.75%) when applied to DSCs, but an enhanced conversion efficiency (*η* = 2.80%) when applied to CdSe-based QDSCs.

On account of the limited fossil fuel quantities in the earth’s surface, investigations into clean and renewable energy sources have been intensively performed for decades, with solar energy currently being considered as one of the most promising candidates to replace conventional energy sources. With the goal of efficiently using solar energy, various kinds of photovoltaics such as Si and thin film solar cells were proposed. Since 1991, after the pioneering report by Brian O’Regan and Michael Grätzel, mesoscopic dye-sensitized solar cells (DSCs) have attracted great attention due to their favorable characteristics: high performance and reliability, applicability of varied materials and designs, low manufacturing cost, and environmental compatibility^1^,^2^,^3^,^4^. In addition, quantum dot-sensitized solar cells (QDSCs), in which semiconducting quantum dots (QDs) are utilized as the light-harvesting units, also have been intensively studied due to QDs’ attractive properties, such as the ability for band energy tuning, high absorption coefficients, and the multiple exciton generation effect^5^,^6^,^7^,^8^.

Ruthenium complexes^9^,^10^ and chalcogenide nanoparticles (such as CdS, CdSe, and PbS)^5^,^6^,^11^,^12^ have been employed as typical sensitizers for DSCs and QDSCs, respectively. For the construction of highly efficient mesoscopic sensitized solar cells, the redox couple in the electrolyte should efficiently quench photogenerated holes in these dyes or QDs, and also be efficiently reduced at the counter electrodes (CEs) in the device^13^,^14^. Until now, the iodide (I^−^/I_3_^−^) and polysulfide (S^2−^/S_n_^2−^) redox couples have been utilized as conventional redox electrolytes for DSCs and QDSCs, respectively. Since the CEs play a key role in the reduction of these electrolytes, their electrocatalytic activity and chemical stability significantly affect the final photovoltaic performance^13^,^14^,^15^,^16^,^17^,^18^,^19^,^20^. Although platinum is known as one of the best electrocatalysts for the iodide redox couple, its high cost and rarity stand in the way of its practical use. Due to these problems, various low cost materials based on carbon materials and conducting polymers were proposed for alternative CEs in DSCs^16^,^17^,^18^,^19^,^20^. In addition, Pt displays a poor electrocatalytic activity for the polysulfide redox couple in QDSCs due to the chemisorption of the S^2−^ ions on its surface^14^,^21^,^22^. The adsorbed S^2−^ ions reduce the rate of charge transfer at the Pt CE, resulting in a steep decrease in the photocurrent and fill factor of QDSCs. Therefore, copper sulfide (Cu_2_S) CEs were proposed for efficient QDSCs; however, these suffer from sulfurization of the photoanode^15^,^23^.

As alternative materials for DSC CEs, metal nitride materials have been studied, with some showing excellent electrocatalytic properties and stabilities, even comparable to Pt^16^,^20^,^24^,^25^. However, an extremely high temperature is often required for the fabrication of metal nitride CEs, leading to significant increases in the manufacturing cost. Herein, we prepared nickel nitride electrocatalysts by reactive sputtering of nickel under a N_2_ atmosphere at room temperature. This facile and reliable method led to the formation of a Ni_2_N film with a cauliflower-like nanostructure. The crystal structure and chemical stability of this CE for the iodide and polysulfide redox electrolytes were investigated in detail. In addition, the electrocatalytic activities of the prepared nickel nitride CE were compared to a conventional Pt CE for both the iodide and polysulfide redox electrolytes. Furthermore, the photovoltaic performances of the DSCs and QDSCs employing either the nickel nitride or conventional Pt CEs were examined and compared.

## Results

### Material Characterization

**Figure 1:**
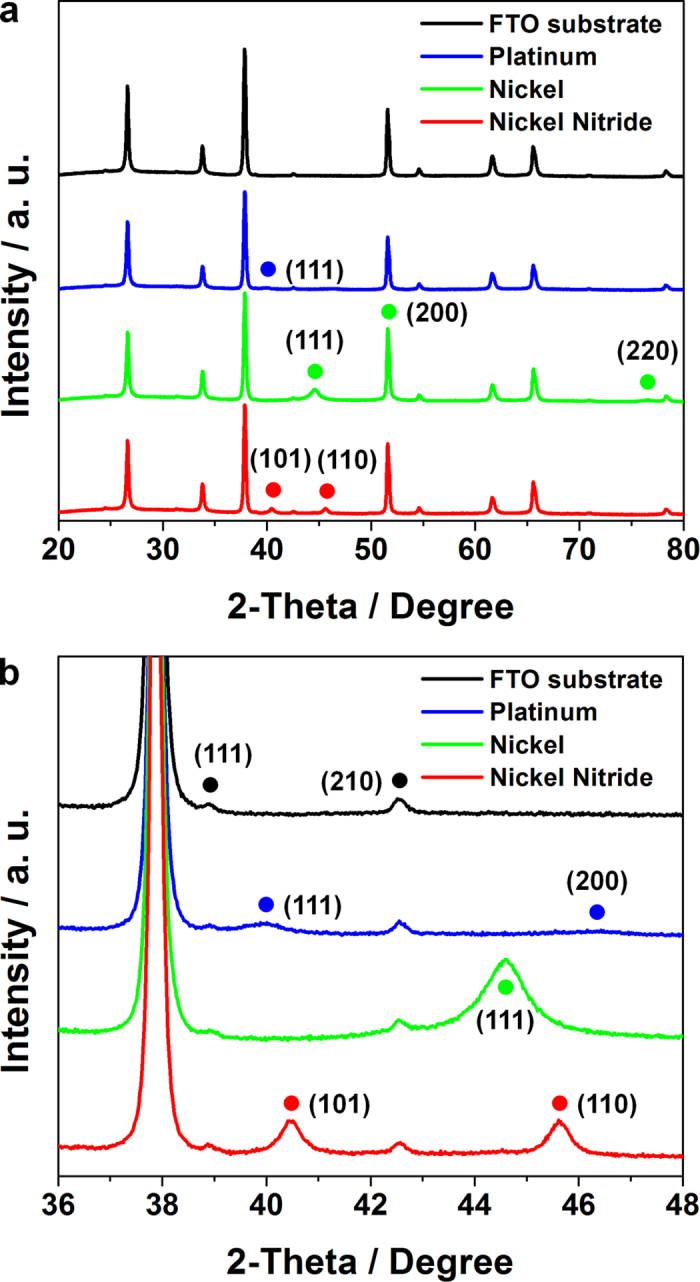
(**a,b**) The XRD spectra of Pt (blue lines), Ni (green lines), and nickel nitride films (red lines) on FTO glasses (black lines), with (**b**) showing a close-up region within (**a**).

**Figure 2:**
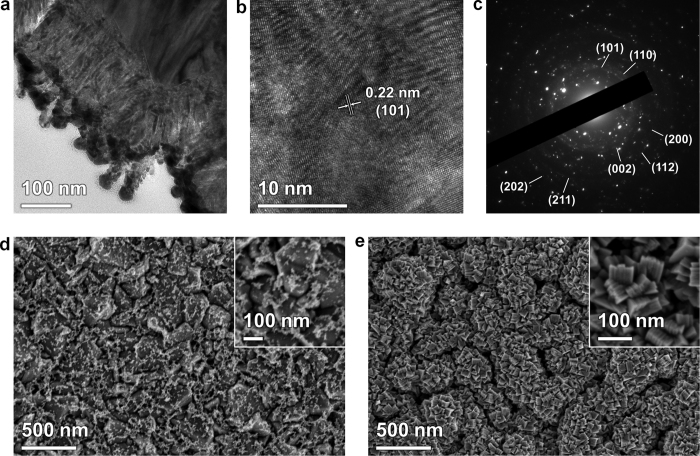
(**a,b**) TEM images at different magnifications and the (**c**) SAED pattern of a nickel nitride film. (**d,e**) SEM image of the (**d**) Pt and (**e**) nickel nitride film on the FTO glasses. The insets of (**d**) and (**e**) are high-magnification images.

Figure 1a,b show the XRD spectra of the FTO glass substrate, the Pt film, the nickel film, and the prepared nickel nitride film deposited on the FTO glasses (Fig. 1b is a close up of a region in Fig. 1a). The peak position of the nickel nitride film was nearly identical to the tetrahedral crystal structure of Ni_2_N^26^. The TEM images were obtained after the preparation of the nickel nitride film using a focused ion beam (FIB) as shown in Fig. 2a–c. The crystal structure of the prepared nickel nitride film was crosschecked by measuring the lattice spacing (Fig. 2b) and assigning the selected area electron diffraction (SAED) pattern (Fig. 2c)^26^. For comparison, the XRD spectrum of a Ni metal film was also obtained, as shown in Fig. 1a,b, with its peak positions assigned to that of metallic Ni (JCPDS 04-0850). As shown in Fig. 1a,1b, the XRD spectrum of the prepared nickel nitride did not display the peaks corresponding to metallic Ni, indicating that the deposited Ni was effectively nitrided by the reactive sputtering process. The Pt film was also prepared as a conventional CE. Its XRD spectrum is in accordance with that of metallic Pt as shown in Fig. 1a,1b (JCPDS 04-0802). As shown in the SEM images (Fig. 2d,e), both the Pt and nickel nitride films were uniformly deposited on the FTO glasses. In particular, the nickel nitride films exhibited a porous and cauliflower-like nanostructure. The thickness of the deposited nickel nitride was about 130 nm, which was estimated from its TEM image showing the film prepared by FIB milling.

**Figure 3:**
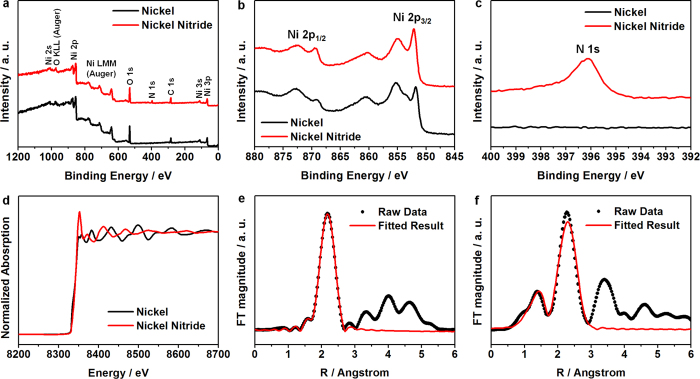
(**a**) XPS spectra of the nickel (black lines) and nickel nitride (red lines) films across a wide scan range for (**b**) the Ni 2p core level peak, and (**c**) the N 1s core level peak. (**d**) The Ni K-edge XANES spectra of nickel and nickel nitride. (**e,f**) The k^3^-weighted Fourier transform of the EXAFS spectra at the Ni K-edge for (**e**) nickel and (**f**) nickel nitride.

The chemical states of the prepared nickel nitride films were examined by XPS measurement. Figure 3a shows the spectra of the nickel and nickel nitride films over a wide scan range. The composition of both films was Ni, O, and C. In addition, N was observed on the nickel nitride film. As shown in Fig. 3b, the metal Ni film exhibited three peaks corresponding to the Ni 2p_3/2_ in the lower binding energy region (850–862 eV), and two peaks corresponding to the Ni 2p_1/2_ in the higher binding energy region (867–875 eV)^27^. The Ni 2p_3/2_ peak at 851.5 eV can be assigned to the Ni-Ni bond^28^,^29^, and the peaks at 855.0 and 860.0 eV can be assigned to the Ni-O bond^27^,^29^, indicating that the film’s surface may be partially oxidized. The nickel nitride film presented nearly the same spectrum as that of the metal Ni film. However, the peak of the Ni-Ni bond shifted to a higher binding energy (851.8 eV) compared to that of the metal Ni film due to the formation of N-Ni bonds^20^. The evidence of the nickel nitridation by the reactive sputtering process was clearly revealed by the N 1s peak shown in Fig. 3c. The nickel nitride film exhibited a clear N 1s peak at 395.8 eV^30^, while the metal Ni film demonstrated no peaks in the same binding energy region.

For further characterization of the prepared nickel nitride’s crystal structure, an X-ray absorption fine structure (XAFS) analysis at the Ni K-edge was performed. From the XANES data (Fig. 3d), it was confirmed that the oxidation state of the nickel nitride film was considerably larger than that of the metal Ni film based on its high-energy shifted edge position and increased white line intensity. This implies a successful nitridation by the reactive sputtering process. The EXAFS was also observed in order to characterize the lattice structure of the nickel nitride film. Figures 3e,f show the Fourier transformed k^3^-weighted EXAFS spectra of the metal Ni and nickel nitride, with the fitted results based on the Kaiser-Bessel window function with d*k* = 1.0 and d*R* = 0.5. The R-factor for the EXAFS fitting of the metal Ni and nickel nitride films was 0.0017 and 0.0196, respectively. The lattice structure of the Ni metal is known to be face centered cubic, while the nickel nitride is comprised of Ni atoms in a body centered tetragonal structure with N atoms located at the two opposite faces of the tetragonal unit cell. In the metal Ni film, the Ni-Ni distance was 2.483 Å from the fitted results. However, the Ni-Ni interatomic distances were much larger in the nickel nitride, which were 2.699 and 2.800 Å, and the Ni-N distance was 1.972 Å. The lattice constants of the nickel nitride, based on the EXAFS results, were a = b = 2.800 and c = 3.686.

### Electrocatalytic Activity and Stability of the Nickel Nitride Electrodes

**Figure 4:**
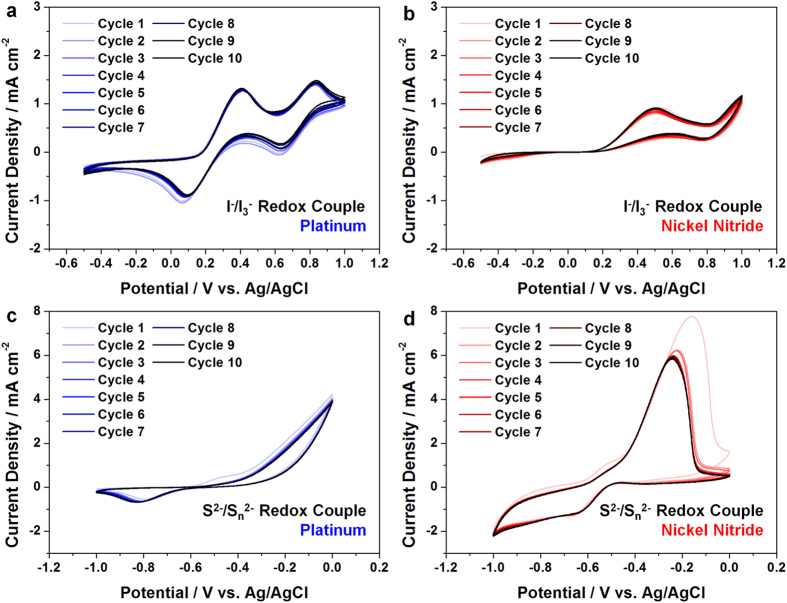
Cyclic voltammograms (CVs) of (**a**) Pt and (**b**) nickel nitride electrodes for the iodide redox couple. CVs of (**c**) Pt and (**d**) nickel nitride electrodes for the polysulfide redox couple.

The electrocatalytic activity and stability of the nickel nitride electrodes were compared with those of conventional Pt electrodes by CV analyses, as shown in Fig. 4. Figure 4a shows two clear pairs of redox potentials for the Pt electrodes with the iodide redox couple. The more positive and negative pairs correspond to the redox reaction of I_2_/I_3_^−^and I^−^/I_3_^−^, respectively^31^. On the other hand, the nickel nitride electrodes did not exhibit clear pairs of redox potentials for the iodide redox couple, and the redox current density was considerably lower compared to the Pt electrodes, as shown in Fig. 4b. However, these electrocatalytic behaviors were reversed for the polysulfide redox couple. While the Pt electrodes did not display remarkable performance for the polysulfide redox couple, the nickel nitride electrodes showed clear redox potentials with a significantly higher current density^32^, as shown in Fig. 4c,4d. These results imply that the nickel nitride electrodes have an inferior electrocatalytic activity for the iodide redox couple compared to the conventional Pt electrodes; however, they have a superior electrocatalytic activity for the polysulfide redox couple. The nickel nitride electrodes maintained the intensity and shape of their CV curves over 10 cycles for both redox couples, indicating that they are electrochemically stable for both redox couples.

**Figure 5:**
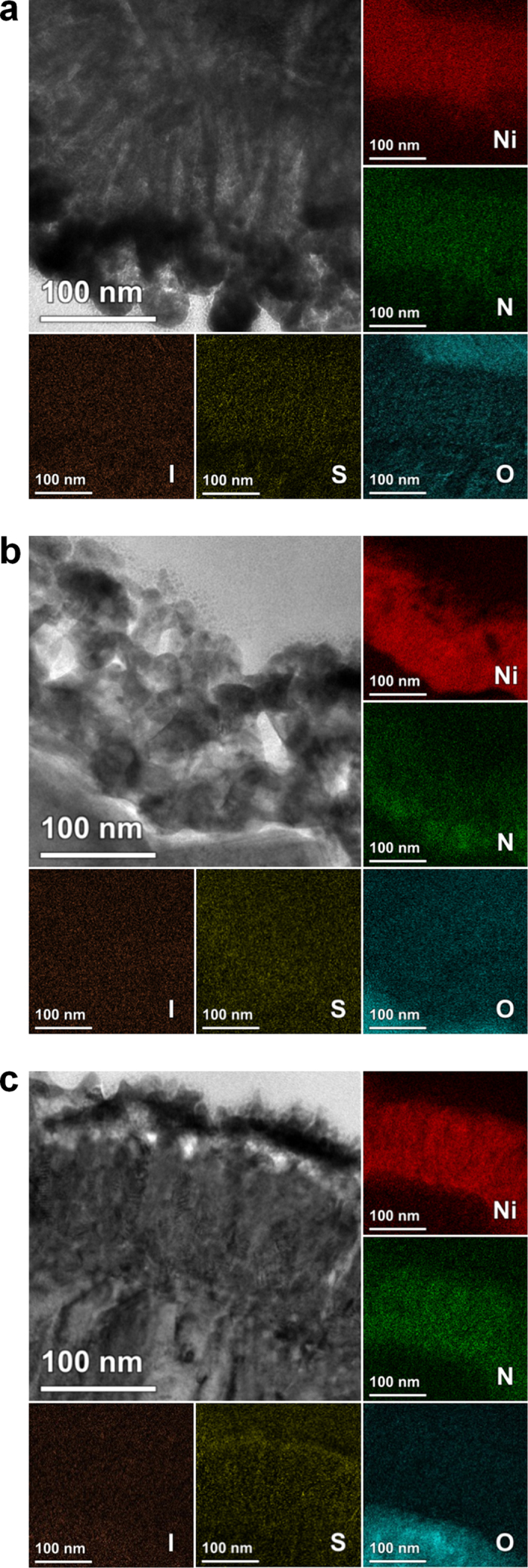
TEM images and elemental EF-TEM maps of the nickel nitride electrodes (**a**) before and (**b,c**) after 10 CV cycles in the iodide and polysulfide redox electrolytes.

From Fig. 4d, relatively large drop in electrocatalytic activity after the first CV cycle is observed. Since this might be attributed to the interaction between the nickel nitride and the polysulfide electrolyte, the electrochemical stability of the nickel nitride electrode was further examined by comparing its crystal structure and chemical states before and after the 10 CV cycles. Supplementary Fig. 1a,b compare the XRD spectra of the nickel nitride electrode before and after 10 CV cycles. It was confirmed that the crystal structure was maintained after multiple CV cycles for both electrolytes (the iodide and polysulfide redox electrolytes), indicating that the nickel nitride was not chemically dissolved in either electrolyte. The chemical states were examined by elemental energy filtered (EF)-TEM maps after the sampling by using FIB (Fig. 5a–c). For this analysis, the electrodes were washed with acetonitrile or DI water after the CV cycles in the iodide or polysulfide electrolyte, respectively, in order to remove remaining redox species on the surface. As shown in Fig. 5, the distributions of Ni and N were preserved after multiple CV cycles in both electrolytes. In addition, iodine (I) was not detected after 10 CV cycles in the iodide electrolyte, indicating that the iodide ions did not adsorb on or chemically react with the nickel nitride. However, sulfur (S) was detected in the electrode after 10 CV cycles in the polysulfide electrolyte. This indicates that the sulfide ions in the electrolytes adsorbed on or chemically reacted with the nickel nitride. The chemical interaction between the nickel nitride and sulfide ions was also confirmed by comparing the XPS spectra of the nickel nitride electrode before and after 10 CV cycles in the polysulfide electrolyte (Supplementary Fig. S2). Compared to the Ni 2p_3/2_ peak of the Ni-Ni bond for the as-prepared electrode (at a binding energy of 851.8 eV), that of the electrode after 10 CV cycles shifted considerably to a higher binding energy (853.3 eV), inferring that the sulfide ions chemically reacted with the nickel nitride^33^. Furthermore, the Ni 2p_3/2_ peaks of the Ni-O bond for the as-prepared electrode (at binding energies of 854.7 and 859.8 eV) became indistinct after 10 CV cycles. This may be due to the sulfide ions substituting for the oxygen atoms at the electrode’s surface. The chemical bond of the Ni-S was clearly detected by the S 2p peak (at a binding energy of 161.5 eV) for the electrodes after 10 CV cycles^33^. Given that XPS is a technique chiefly for surface analysis, and that the bulk distributions of the Ni and N species were maintained after numerous CV cycles (as confirmed by the elemental EF-TEM maps), it can be concluded that the bulk structure of the nickel nitride electrodes is chemically stable toward the polysulfide electrolyte; however, a chemical reaction is possible between the electrode surface and the sulfide ions. Recently, Kim *et al.* reported that the nickel sulfide (NiS) films displayed a high electrocatalytic activity toward the polysulfide redox couple^34^. Considering these results, the chemical reaction with sulfide ions at the electrode surface may not deteriorate the electrocatalytic activity of the nickel nitride toward the polysulfide electrolyte. In addition, as already shown in Fig. 3, the chemical bond between Ni and O was detected by XPS; however, these oxygen species were not detected by the elemental EF-TEM maps (Fig. 5a–c) except in the region of the FTO glass, implying that the prepared nickel nitride electrode was oxidized only at the surface.

**Figure 6:**
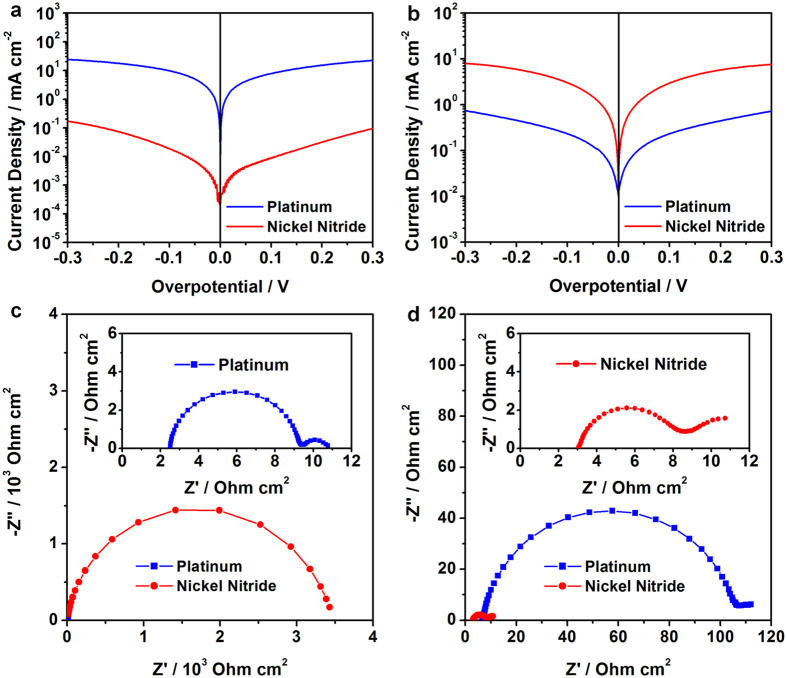
Tafel polarization curves of symmetric dummy cells with Pt (blue lines/squares) or nickel nitride (red lines/circles) electrodes for (**a**) iodide and (**b**) polysulfide electrolytes. Impedance spectra of the symmetric dummy cells for (**c**) iodide and (**d**) polysulfide electrolytes, with the insets showing the enlarged spectra.

The electrocatalytic activities of the Pt and nickel nitride electrodes were reconfirmed by Tafel polarization measurements^16^, as shown in Fig. 6a,b. For these measurements, we prepared symmetric dummy cells with the Pt or nickel nitride electrodes. The Tafel polarization curves reveal a logarithmic current density (*J*) as a function of voltage (*V*). A larger slope of the Tafel curve means a higher exchange current density (*J*_0_), and this indicates superior electrocatalytic activity. As shown in Fig. 6a, the Pt electrode showed a significantly higher *J*_0_ for the iodide electrolyte compared to that of the nickel nitride electrode. However, this situation was reversed for the polysulfide electrode, shown in Fig. 6b, with the nickel nitride electrode showing a considerably higher *J*_0_ than the Pt electrode. These results suggest that the electrocatalytic activity of the nickel nitride electrode is inferior for the iodide redox couple, but superior for the polysulfide electrolyte compared to the Pt electrode, which is coincident with the CV data. In order to quantitatively compare the electrocatalytic activities of the Pt and nickel nitride electrodes, we obtained impedance spectra from the same symmetric dummy cells, shown in Fig. 6c,6d. We used the equivalent circuit model, comprising a series resistance (*R*_s_), an impedance at the electrolyte/electrode interface (*R*_ct_ and CPE), and a finite Warburg impedance (*W*_s_) related to the electrolyte diffusion (Supplementary Fig. S3)^17^,^35^. *R*_ct_ is related to the charge transfer at the interface between the electrode and the electrolyte. CPE stands for “constant phase element”, which usually replaces a capacitor in the equivalent circuit, is attributed to the electrodes’ roughness^36^. From the CPE, a double-layer capacitance (*C*_dl_) can be evaluated. These two values (*R*_ct_ and *C*_dl_), which are related to the electrocatalytic activity, were obtained after fitting the impedance spectra by the ZView software, as listed in Table 1. For the iodide redox couple, the nickel nitride electrode had a considerably larger *R*_ct_ compared to the Pt electrode, indicating its inferior electrocatalytic activity, because the *R*_ct_ is related to the *J*_0_ by *J*_0_=(RT)/(nF*R*_ct_)^17^,^35^. In contrast, for the polysulfide redox couple, the nickel nitride electrode exhibited significantly increased *C*_dl_ as well as the reduced *R*_ct_, implying numerous electrochemical reaction sites. Significantly enhanced electrocatalytic activity of the Ni nitride can be attributed to fast charge transfer and numerous electrochemical reaction sites. From these results, it can be expected that the nickel nitride CEs may be suitable for the QDSCs rather than for the DSCs.

### Application of the Nickel Nitride Electrodes to the Mesoscopic Dye- or QD-Sensitized Solar Cells

**Figure 7:**
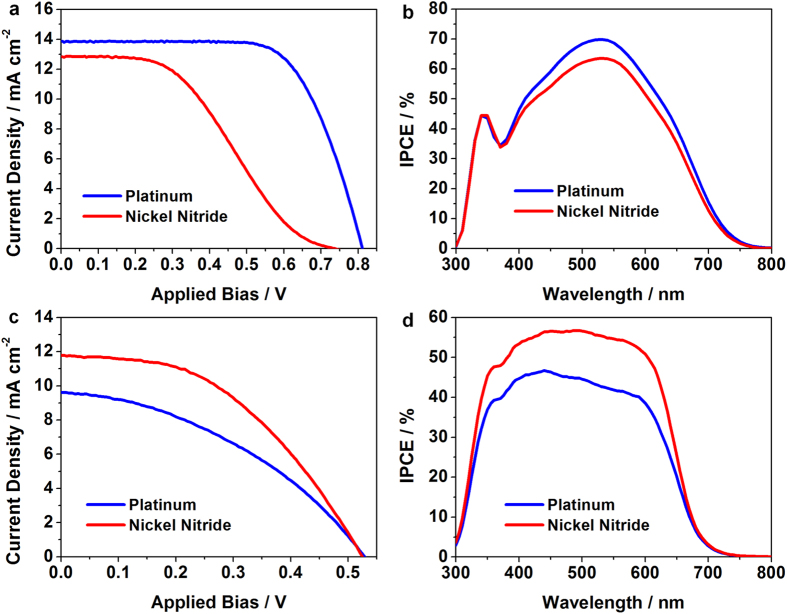
(**a**) Photocurrent density-voltage (*J-V*) characteristics under illumination (light intensity: 100 mW cm^−2^, AM 1.5G filter) and (**b**) IPCE spectra of the DSCs with Pt (blue lines) or nickel nitride (red lines) CEs. (**c**) Photocurrent density-voltage (*J-V*) characteristics under illumination (light intensity: 100 mW cm^−2^, AM 1.5G filter) and (**d**) IPCE spectra of the QDSCs with each type of CE. The DSCs and QDSCs utilized the iodide and polysulfide redox electrolyte, respectively.

Finally, the prepared nickel nitride electrodes were introduced as CEs to the mesoscopic DSCs and QDSCs. Figure 7 shows the photocurrent density-voltage (*J*-*V*) characteristics and the IPCE spectra of the DSCs and QDSCs with either the Pt or nickel nitride CEs. The photovoltaic performance parameters are listed in Table 2. As listed in Table 2, the DSC with the nickel nitride CE exhibited a lower conversion efficiency (*η, η* = 3.75%) than that with the conventional Pt CE (*η* = 7.62%). This lower efficiency was mainly due to a poor fill factor, which is closely related to the inferior electrocatalytic activity of the nickel nitride electrode for the iodide redox electrolyte^16^. The lower short-circuit current density (*J*_sc_) of the nickel nitride CE was also confirmed by the IPCE spectra (Fig. 7b). However, when applied in the QDSCs, the nickel nitride CE exhibited significantly enhanced cell performances over the Pt CE. The *J*_sc_ and fill factor were all enhanced for the QDSC with the nickel nitride CE, resulting in a significantly increased conversion efficiency (by 39%) compared to that with the Pt CE. The far higher *J*_sc_ was also confirmed by the IPCE spectra (Fig. 7d). This enhanced photovoltaic performance was attributed to the excellent electrocatalytic activity of the nickel nitride CE for the polysulfide redox electrolyte. This trend of photovoltaic performances accorded well with the CV, Tafel polarization, and impedance data. Also, the elongation of light pathways by reflection was slightly larger in the cells employing nickel nitride CEs than those with Pt CEs (Supplementary Fig. S4), and this also seems to have contributed to a more efficient light utilization. These results suggest that the nickel nitride CE can be utilized promisingly for the QDSCs employing the polysulfide electrolyte. In this work, we investigated the electrocatalytic activity of the nickel nitride CE only for the iodide and polysulfide redox couples; meanwhile, there are several alternative redox couples for the mesoscopic dye- and QD-sensitized solar cells, including the cobalt-based^2^,^37^ or disulfide/thiolate^38^ redox couples. Further studies on examining the electrocatalytic activity of the nickel nitride CE for these redox couples may be worthwhile as future work.

## Discussion

We developed nickel nitride CEs for application in mesoscopic dye- and QD-sensitized solar cells through a new process, that of reactive sputtering at room temperature. In-depth studies revealed that this facile and reliable method led to the formation of a Ni_2_N film with a cauliflower-like nanostructure and tetrahedral crystal lattice. In addition, the prepared nickel nitride electrodes showed excellent chemical stability toward both iodide and polysulfide redox electrolytes. Compared to conventional Pt electrodes, the nickel nitride electrodes displayed an inferior electrocatalytic activity for the iodide redox electrolyte; however, they revealed a significantly enhanced electrocatalytic activity for the polysulfide redox electrolyte. In DSCs employing the iodide redox electrolyte, the nickel nitride CEs showed lower conversion efficiency than the conventional Pt CEs. However, when applied in QDSCs employing the polysulfide redox electrolyte, the nickel nitride CEs demonstrated significantly enhanced conversion efficiency over the conventional Pt CEs. These results infer that reactive sputtering is a reliable method for the preparation of efficient nickel nitride CEs at room temperature, and these prepared nickel nitride CEs should be promising electrocatalysts in the mesoscopic QD-sensitized solar cells.

## Methods

### Fabrication of the Pt and Nickel Nitride CEs

Pt CEs were prepared by thermal decomposition of a commercial Pt precursor-containing paste (PT1, Dyesol Ltd.) applied using the doctor blading method. The paste was coated onto FTO glass (TEC-8, Pilkington) and then thermally treated in air at 450 ºC for 30 min. Nickel nitride CEs were fabricated by a RF sputtering of nickel (Ni) onto the FTO glass for 3 h. During this deposition, the chamber was filled with 80 mTorr N_2_ gas, and the reactive power of 150 W was maintained with a 13.56 MHz frequency. FTO glasses with only Ni deposited on the surface were also prepared for material characterization, using an identical method to the nickel nitride electrodes, except that the sputtering was conducted under an 80 mTorr Ar atmosphere.

### Preparation of the Iodide and Polysulfide Redox Electrolytes

All of the chemicals mentioned below were purchased from Sigma-Aldrich and were used without further purification. Two types of iodide (I^−^/I_3_^−^) and polysulfide (S^2−^/S_n_^2−^) redox electrolytes were prepared, some for the measurement of electrocatalytic activity by cyclic voltammetry (CV), and the others for the optimized photovoltaic device operation. For the CV measurements, the iodide electrolytes were prepared by mixing 10 mM LiI, 1 mM I_2_, and 0.1 M LiClO_4_ in acetonitrile, and the polysulfide electrolytes were prepared by mixing 0.1 M Na_2_S and 0.1 M S in deionized (DI) water. For the symmetric cell analyses and device operation, the iodide electrolytes were prepared by mixing 0.6 M 1-butyl-3-methylimidazolium iodide, 30 mM I_2_, 0.1 M guanidinium thiocyanate, and 0.5 M 4-*tert*-butylpyridine in a mixture of acetonitrile and valeronitrile (volumetric ratio = 85 : 15), and the polysulfide electrolytes were prepared by mixing 0.5 M Na_2_S, 2 M S, and 0.2 M KCl in a mixture of DI water and methanol (volumetric ratio = 3 : 7).

### Preparation of the Working Electrodes and Cell Assembly

For the preparation of the TiO_2_ working electrodes, first a dense TiO_2_ blocking layer was coated onto the FTO glasses by dipping them in a 40 mM TiCl_4_ aqueous solution at 70 ºC for 30 min. Then, a colloidal TiO_2_ paste (DSL 18NR-T, Dyesol Ltd.) was deposited on these pretreated FTO glasses by the doctor blading method, followed by annealing at 500 ºC for 30 min. The annealed electrodes were treated again with a 16 mM TiCl_4_ aqueous solution at 70 ºC for 30 min. For the dye-sensitization, the prepared TiO_2_ electrodes were immersed in an ethanol solution of 0.5 mM *cis*-bis(isothiocyanato)bis(2,2`-bipyridyl-4,4-dicarboxylic acid) ruthenium (II) (N719 dye, Ohyoung Industrial Co.) for 48 h. Meanwhile, the CdSe quantum dot-sensitization was carried out by a conventional successive ionic layer adsorption and reaction (SILAR) method in a glove box filled with argon gas^39^. The prepared TiO_2_ electrodes were dipped into a 30 mM Cd(NO_3_)_2_ solution in ethanol for 1 min, followed by rinsing with ethanol. The electrodes were then dipped into a Na_2_Se in ethanol solution for 1 min, followed by rinsing with ethanol. The Na_2_Se solution was prepared using a 30 mM SeO_2_ and 60 mM NaBH_4_ solution in ethanol, following a previous paper^39^. Finally, the ZnS was coated on the surface of QD-sensitized TiO_2_ photoanode by immersion of electrode into 0.5 M Zn(CH_3_COO)_2_ and then in 0.5 M Na_2_S solution for 5 min each. The dye- or quantum dot-sensitized working electrodes were assembled with the CEs using a thermoplastic sealant (Surlyn, Dupont) with a 60 μm thickness. The electrolytes were injected into the assembled cell through a pre-drilled hole. Symmetric cells for the evaluation of electrocatalytic activity were fabricated with exactly the same method used for DSC and QDSC assembly except for that the two identical Pt or nickel nitride electrodes were used.

### Characterization of Materials, Electrochemical Analysis, and Photovoltaic Performance Evaluation

Scanning electron microscopy (SEM) analyses were conducted using a Carl Zeiss SUPRA 55VP and a Carl Zeiss AURIGA. A transmission electron microscopy (TEM) analysis was performed with a JEOL JEM-2100F after sampling by a focused ion beam (FIB) milling using a SMI3050SE, SII Nanotechnology. X-ray diffraction (XRD) patterns were obtained using a Rigaku D-MAX2500-PC. An X-ray photoelectron spectroscopy (XPS) analysis was conducted using a Thermo Fisher Scientific SIGMA PROBE. X-ray absorption near edge structure (XANES) and extended X-ray absorption fine structure (EXAFS) spectra were obtained by synchrotron measurement at the 8C beamline of the Pohang Accelerator Laboratory (PAL, Republic of Korea). CV analysis was conducted using a potentiostat (PGSTAT128N, Metrohm Autolab) with a Pt mesh counter electrode and Ag/AgCl reference electrode. The scan rate for the CV analysis was 50 mV s^−1^. Tafel polarization and electrochemical impedance spectroscopy measurement were done by using Metrohm Autolab PGSTAT128N and Zahner IM6 workstation, respectively, after the preparation of symmetric cells. Photovoltaic properties were characterized by solar simulators (XIL model 05A50KS source measure units, SERIC Ltd. for DSCs and PEC-L11, Peccell Technologies Inc. for QDSCs) under a 1 sun condition (AM 1.5G with an incident light intensity of 100 mW cm^−2^), which was verified by a National Institute of Advanced Industrial Science and Technology (AIST, Japan) calibrated Si reference solar cell. The active area of the dye- or QD-sensitized TiO_2_ film was 0.16 cm^2^. Prior to the photovoltaic measurement, a black aperture mask covered the devices in order to avoid overestimation of the conversion efficiency^40^. The incident photon-to-current efficiencies (IPCE) of the DSCs and QDSCs were measured using a QEX7 (PV Measurements Inc.) or PEC-S20 (Peccell Technologies Inc.), respectively.

## Additional Information

**How to cite this article**: Kang, J. S. *et al.* Reactively sputtered nickel nitride as electrocatalytic counter electrode for dye- and quantum dot-sensitized solar cells. *Sci. Rep.* **5**, 10450; doi: 10.1038/srep10450 (2015).

## Supplementary Material

Supplementary Information

## Figures and Tables

**Table 1:** Parameters determined by fitting the impedance spectra of symmetric dummy cells with Pt and nickel nitride electrodes.

**Electrode**	**Electrolyte**	***R***_**ct**_**/ Ω cm**^**2**^	***C***_**dl**_**/ μF cm**^**– 2**^
Pt	Iodide	3.37	64.2
Nickel nitride	Iodide	1719.50	73.6
Pt	Polysulfide	51.80	266.8
Nickel nitride	Polysulfide	2.49	3192.0

**Table 2:** Summary of *J-V* characteristics for DSCs and QDSCs employing Pt and nickel nitride CEs.

**Electrode**	***V***_**oc**_ **/ V**	***J***_**sc**_ **/ mA cm**^**–2**^	***FF*** **/ %**	***η*** **/ %**
DSCs with Pt CEs	0.812(0.812 ± 0.003)	13.8(13.7 ± 0.4)	68.0(66.9 ± 1.6)	7.62(7.41 ± 0.19)
DSCs with nickel nitride CEs	0.738(0.738 ± 0.011)	12.8(12.5 ± 0.4)	39.7(38.8 ± 1.1)	3.75(3.58 ± 0.14)
QDSCs with Pt CEs	0.529(0.507 ± 0.019)	9.6(8.8 ± 0.8)	39.4(38.4 ± 0.9)	2.01(1.75 ± 0.23)
QDSCs with nickel nitride CEs	0.525(0.518 ± 0.006)	11.8(11.6 ± 0.2)	45.2(44.7 ± 0.5)	2.80(2.76 ± 0.04)

The statistical data (average ± standard deviation) of three samples are presented in parentheses.
