# Supplementary material for: Reactively sputtered nickel nitride as electrocatalytic counter electrode for dye- and quantum dot-sensitized solar cells
Source: Sci Rep. 2015 May 21;5:10450. doi: 10.1038/srep10450 (PMC5386249; doi:10.1038/srep10450)
Supplement: Supplementary Information [file srep10450-s1.doc]

Supplementary Information

**Reactively sputtered nickel nitride as electrocatalytic counter electrode for dye- and quantum dot-sensitized solar cells**

Jin Soo Kang^1,2^,*, Min-Ah Park^3,4^,*, Jae-Yup Kim^4^,*, Sun Ha Park^5^, Dong Young Chung^1,2^,

Seung-Ho Yu^1,2^, Jin Kim^1,2^, Jongwoo Park^4^, Jung-Woo Choi^1,2^, Kyung Jae Lee^1,2^, Juwon Jeong^1,2^,

Min Jae Ko^4,6,7^, Kwang-Soon Ahn^8^ & Yung-Eun Sung^1,2^

^1^Center for Nanoparticle Research, Institute for Basic Science (IBS), Seoul 151-742, Republic of Korea.

^2^School of Chemical and Biological Engineering, Seoul National University, Seoul 151-742, Republic of Korea.

^3^Department of Nanomaterials Science and Engineering, Korea University of Science and Technology, Daejeon 305-350, Republic of Korea.

^4^Photo-electronic Hybrids Research Center, Korea Institute of Science and Technology (KIST), Seoul 136-791, Republic of Korea.

^5^Samsung SDI Materials Devision, OLED Development Group 1, Uiwang 437-711, Republic of Korea.

^6^Green School, Korea University, Seoul 136-701, Republic of Korea.

^7^KU-KIST Graduate School of Converging Science and Technology, Korea University, Seoul 136-701, Republic of Korea.

^8^School of Chemical Engineering, Yeungnam University, Gyeongsan 712-749, Republic of Korea.

*These authors contributed equally to this work.

CORRESPONDING AUTHORS

Yung-Eun Sung (e-mail: ysung@snu.ac.kr)

Kwang-Soon Ahn (e-mail: kstheory@ynu.ac.kr)

Min Jae Ko (e-mail: mjko@kist.re.kr)


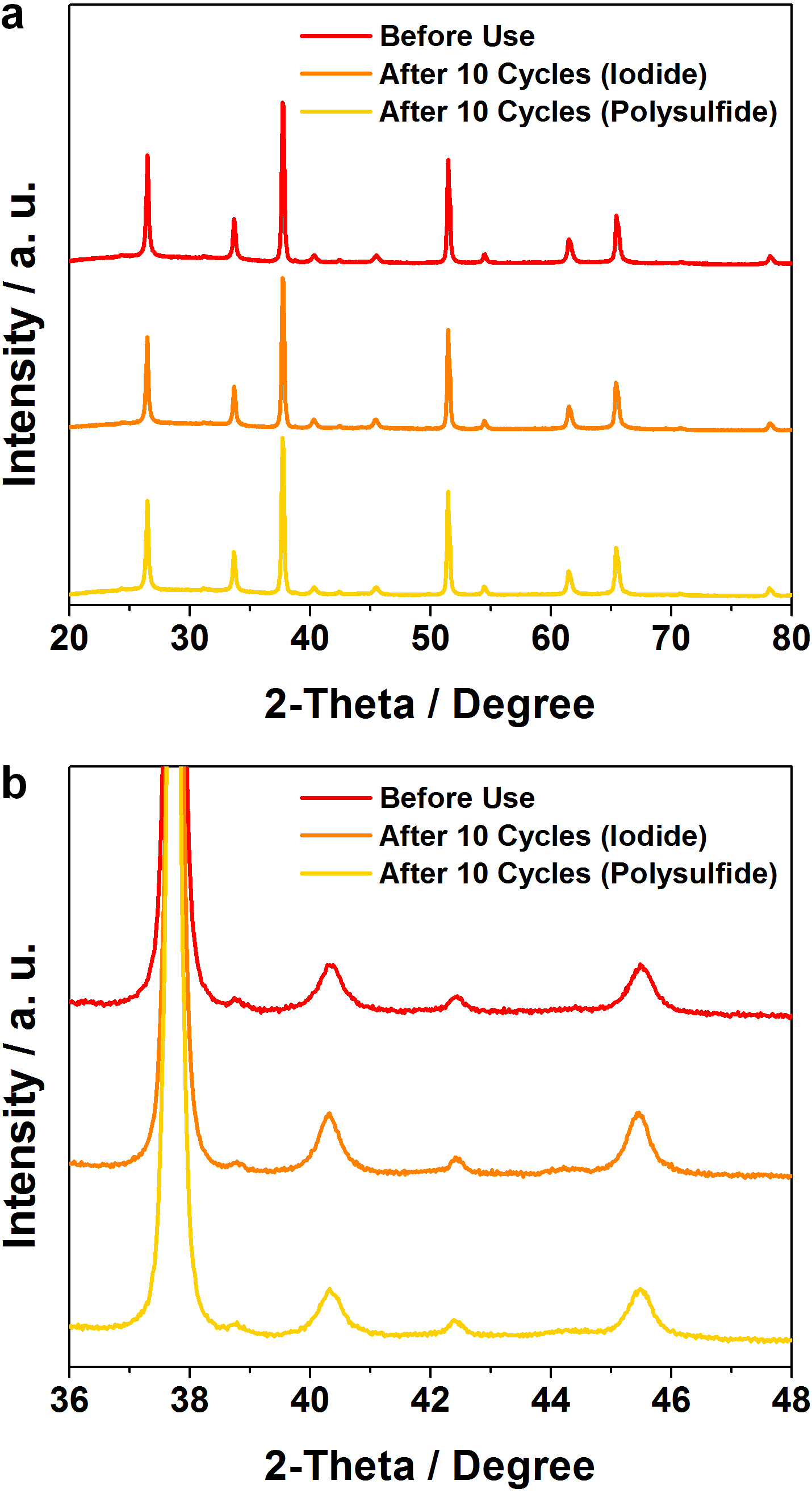


**Figure S1.** XRD spectra of nickel nitride electrodes before (red lines) and after 10 CV cycles in the iodide (orange lines) and polysulfide electrolytes (yellow lines), with (b) being a close-up of a region in (a).

**
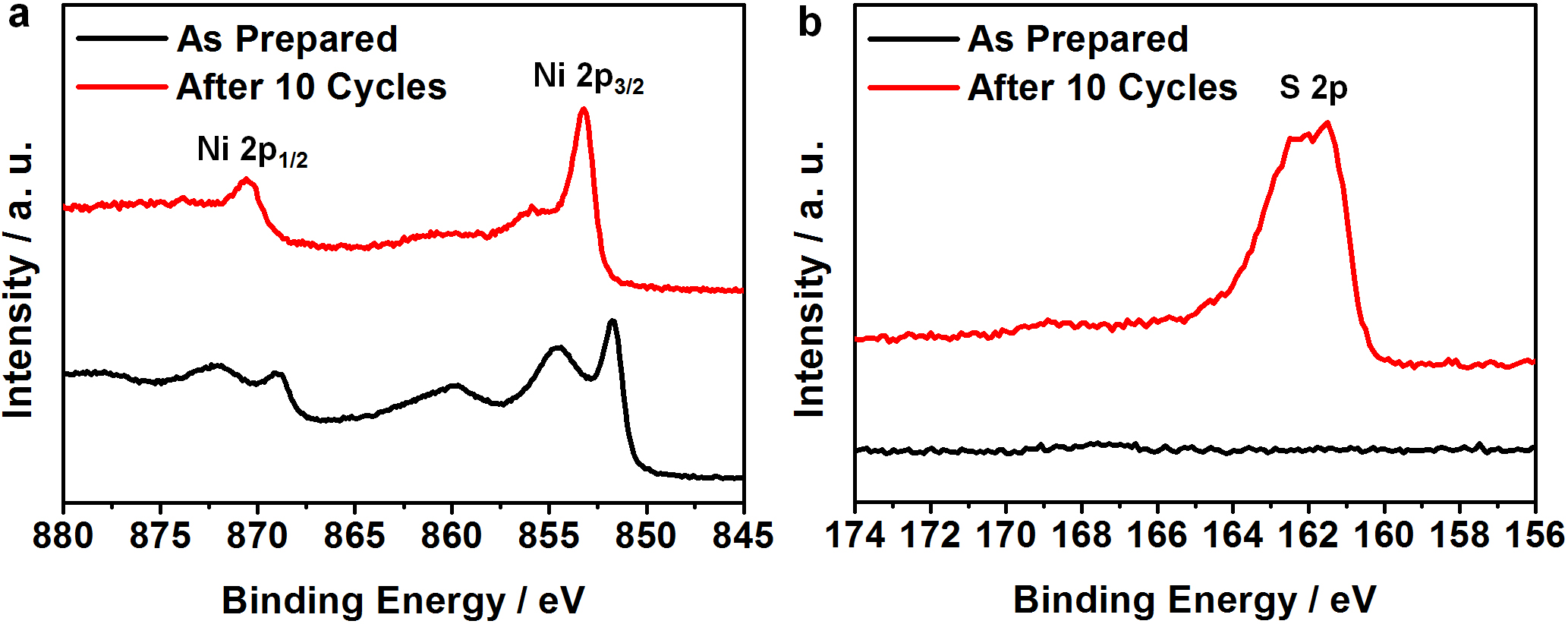
**

**Figure S2.** (a) Ni 2p and (b) S 2p core level XPS spectra of nickel nitride electrodes before and after 10 CV cycles in the polysulfide electrolytes.


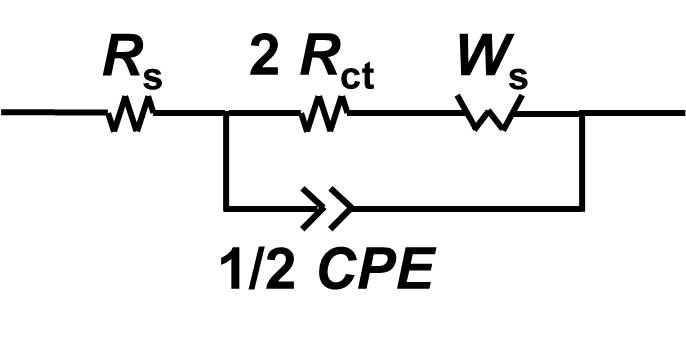


**Figure S3.** The equivalent circuit model for the impedance spectra of symmetric dummy cells shown in Fig. 6c and 6d.


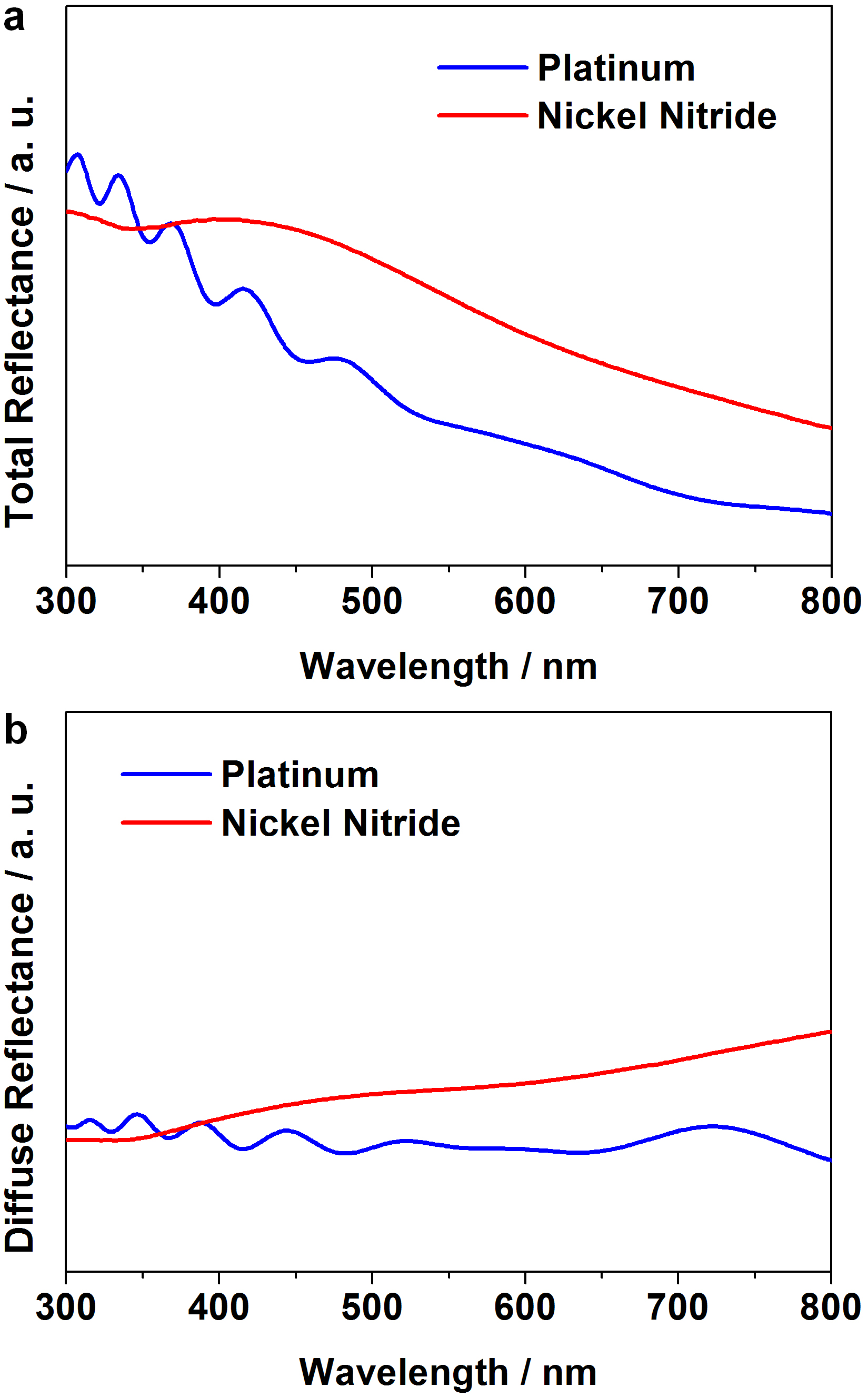


**Figure S4.** (a) Total reflectance and (b) diffuse reflectance spectra of platinum and nickel nitride counter electrodes.
